# Oncogenic BRCA1,2 Mutations in the Human Lineage—A By-Product of Sexual Selection?

**DOI:** 10.3390/biomedicines12010022

**Published:** 2023-12-21

**Authors:** Tatyana V. Korneenko, Nikolay B. Pestov

**Affiliations:** 1 Group of Cross-Linking Enzymes, Shemyakin-Ovchinnikov Institute of Bioorganic Chemistry, Moscow 117997, Russia; 2 Institute of Biomedical Chemistry, Moscow 119121, Russia; 3 Chumakov Federal Scientific Center for Research and Development of Immune-and-Biological Products, Moscow 108819, Russia

**Keywords:** breast cancer, estrogens, evolution, sexual selection, mastectomy

## Abstract

In this review, we discuss the long-known problem of tissue-specific carcinogenesis in BRCA1 and BRCA2 mutation carriers: while the genes are expressed ubiquitously, increased cancer risk is observed mostly in the breast and ovaries, and to a much lesser extent, in some other tissues such as the prostate or pancreas. We reevaluate hypotheses on the evolutionary origin of these mutations in humans. Also, we align together the reports that at least some great apes have much lower risks of epithelial cancers in general and breast cancer in particular with the fact that humans have more voluminous breast tissue as compared to their closest extant relatives, particularly chimpanzees and bonobos. We conjecture that this disparity may be a consequence of sexual selection, augmented via selection for enhanced lactation. Further, we argue that there is an organ-specific enigma similar to the Peto paradox: breast cancer risk in humans is only minimally correlated with breast size. These considerations lead to the hypothesis that, along with the evolutionary development of larger breasts in humans, additional changes have played a balancing role in suppressing breast cancer. These yet-to-be-discovered mechanisms, while purely speculative, may be valuable to understanding human breast cancer, though they may not be exclusive to the mammary gland epithelial cells. Combining these themes, we review some anti-carcinogenesis preventive strategies and prospects of new interventions against breast cancer.

## 1. Introduction

Breast size in humans is a sexually dimorphic trait. Also, it is well established that breast size is among the features that distinguish humans from their closest relatives among extant great apes.

However, it is not clear whether it was shaped via sexual or natural selection. It is tempting to speculate that sexual selection is the most probable explanation for this trait; indeed, the estimations of female breast size preferences among males strongly support this notion. Also, the relative size of human breasts is considerably larger than in other primate species and empirical research across various human populations has consistently demonstrated a male preference for women with average or larger breast sizes. This preference is linked to perceived indicators of higher reproductive potential, lactation capability, and libido, though this trend is not absolute [1,2,3,4,5,6,7]. Also, there are theories suggesting that sexual selection was predominantly acting on males, whereas natural selection was more significant in females, of which the accumulation of adipose tissue has been a critical factor in determining breast size [8]. Nevertheless, it has been pointed out long ago that evolutionary hypotheses about human breasts are notoriously difficult to test experimentally [9].

The most important question nevertheless is about clarification on whether this evolutionary acquisition came with a significant cost in terms of increased breast cancer (BC) incidence. Therefore, we searched for mostly recent articles devoted to BC molecular mechanisms, as well as for studies on the correlation between BC risk and breast size, BC in primates, and the molecular evolution of BRCA1 and BRCA2 genes.

## 2. Types of Breast Cancer and Molecular Basis behind Current and Classical Views on the Mechanisms of Carcinogenesis in the Mammary Gland

BC is a diverse group of cancers with distinct molecular and clinical features. Despite this diversity, their shared tissue origin sets margins to key properties crucial for the onset and progression of BC.

### 2.1. Human BC

Clinically, BC is categorized into three grades: low, intermediate, and high. From a pathohistological standpoint, keratins serve as valuable markers in BC: downregulation of KRT15,14,17,5 and upregulation of KRT18,8 [10], together with cell division marker Ki67. Four distinct subtypes of BC are usually noted, where the ER (estrogen receptor), PgR (progesterone receptor), and HER2 (a membrane protein tyrosine kinase) molecular markers are especially helpful for classification, with an increasingly worse prognosis from 1 to 4 [11,12]:

(1) Luminal A subtype: ER and/or PgR-positive, HER2-negative, and low Ki67;
(2) Luminal B subtype: ER and/or PgR-positive, HER2-positive or negative, and high Ki67;
(3) HER2-enriched subtype: frequently features not only HER2+ positivity but also its gene amplification, lacks ER or PgR expression;
(4) Basal-like triple negative BC (TNBC)–lacking HER2, ER and PgR, with frequent p53 mutations. TNBC is generally high grade with the poorest prognosis of all subtypes. This subtype is more common among younger, premenopausal women and is more prevalent in those at a higher genetic risk.

It is necessary to distinguish between familial and sporadic cases of BC. In our review, we are particularly interested in BC associated with inherited germline mutations. BRCA1 loss is not observed in sporadic BC and ovarian cancer (OC), of which the role of BRCA1 in BC appears to be limited to familial cases. While the general lifetime risk for a female to develop BC stands at approximately 12%, this increases to between 47 and 87% for those carrying a deactivating BRCA1 germline mutation. Typically, BRCA1 germline mutations are indicative of so-called Hereditary Breast and Ovarian Cancer (HBOC) syndrome. BC in BRCA1 mutation carriers most frequently resembles the TNBC subtype, whereas BRCA2 deficiency gives multiple varieties with luminal B ER+ being more frequent [11,12].

### 2.2. BC in Species Other than Humans

While mouse models are invaluable for studies on the molecular mechanism of BC genesis and progression [11], rodents are evolutionary distant from humans.

In the broad context of primates, the few well-studied species show that primates are not characterized by a uniquely low occurrence of tumors. However, the most common malignant tumors in these species are neoplasms of the hematopoietic organs, primarily lymphomas and leukemias. Many of these are induced by oncogenic viruses such as the retrovirus C-type STLV-1, similar to the human oncogenic virus HTLV-1 and the EBV-like herpes virus HVP–herpes virus papio, among others [13].

Macaques and baboons exhibit spontaneous mammary ductal hyperplastic and neoplastic lesions in older captive animals with an estimated lifetime incidence of carcinoma of about 6%. Histologically, most of these carcinomas are similar to the human Luminal A type, expressing both ER and PgR, while some tumors are HER-2 positive or TNBC. Yet, nothing similar to BRCA1 and BRCA2 mutations has been observed in these species. Overall, tumorigenesis in these species appears to be understudied, especially because most experimental animals are not kept throughout their entire lifespan, particularly during their postmenopausal years, when most analogous BC cases occur in women [14,15,16,17].

The lower incidence of cancers originating from epithelial tissues, particularly the absence of breast tumors as well as carcinomas of the colon, breast, lung, prostate, stomach, and pancreas, in captive chimpanzees kept in zoos, is remarkable and has been extensively reviewed [18,19,20]. However, this conclusion is based on observations of possibly thousands of chimpanzees, underscoring a significantly reduced frequency of breast cancer (BC) in chimpanzees compared to humans. A recent paper is extremely valuable, reporting that, indeed, it is difficult to detect any incidence of BC in chimpanzees [18]. As a word of caution, we should add, however, that sample sizes in such studies are usually rather small, barely minimal for statistical significance.

Thus, while perfect analogs of human breast cancer (BC) remain elusive in great apes, the incidence of sporadic BC in these primates is ostensibly lower than in humans, with frequencies only estimable as upper limits. Additionally, familial, heritable BC is not yet identified and prospects of its discovery seem bleak due to the limited population sizes.

## 3. Recent Breakthroughs in the Molecular Mechanisms of Breast Cancer Genesis and Progression

A number of remarkable discoveries on the genesis of BC in BRCA1 mutation carriers have been published in recent years. In individuals with BRCA1 mutations, the loss of a functioning BRCA1 allele corresponds with the emergence of lobules marked by the stem/progenitor indicator ALDH1, alongside a discernible absence of epithelial differentiation markers and ER. The disruption of BRCA1 may yield genetically unstable stem/progenitor cells, which then become prime candidates for subsequent carcinogenic events, such as p53 mutations [21].

It is intriguing that breast cancers (BC) in BRCA1 mutation carriers predominantly originate from cells of the luminal lineage. In these individuals, the differentiation processes of breast gland cells within the luminal lineage seem to be downregulated. The levels of BRCA1 mRNA in luminal progenitor cells are markedly higher than in other cell types in both BRCA1 mutation carriers and non-carriers. Despite this, luminal progenitors from BRCA1 mutation carriers exhibit a substantially reduced BRCA1 expression compared to non-carriers. Furthermore, p53, a key driver gene in basal-like tumors, shows significant downregulation in these luminal progenitors [22].

Especially interesting is the identification of an anomalous luminal progenitor cell population in BRCA1 mutation carriers, which has abundant tyrosine kinase KIT [23]. Importantly, when isolated and grown ex vivo, progenitor-enriched cells from BRCA1 mutation carriers demonstrated heightened mitotic activity and a reduced propensity for cell death. Mechanistically, mutations in BRCA1 that impede its E3 ubiquitin ligase activity appear to enhance PLK1 (polo-like serine/threonine protein kinase) activity at prometaphase spindle poles, possibly via the stabilization of HMMR (hyaluronan mediated motility receptor) or other intermediary factors. This increased mitotic activity of PLK1 ultimately leads to the loss of control over the cell division, hindering the descendant cells’ capacity to acquire mature luminal cell characteristics [24].

The role of the microenvironment in cancer progression is gradually being elucidated. Notably, fibroblasts from BRCA1+/mut carriers exhibit a phenotype resembling cancer-associated fibroblasts (CAF) even at the precancerous stage, commonly referred to as the “pre-CAF” phenotype. These fibroblasts show a CAF-like transcriptional signature known to play a role in tumor progression [25].

A recent study on sporadic BC has yielded a particularly exciting finding. Phylogenetic trees, reconstructed to include both cancerous and non-cancerous clones, were utilized to trace the comprehensive history of BC. These trees were developed using single-cell-derived organoids cultivated from patients diagnosed with BC as well as from the breast milk of healthy breastfeeding volunteers. In each of the five cases studied, the phylogenetic analysis predominantly identified one or two expansive clades. Notably, both cancerous and non-cancerous tissues, including those deemed normal, exhibited the recurrence of der(1;16), a chromosomal abnormality commonly associated with Luminal A BC, particularly in invasive lobular types. Intriguingly, cancerous clones often appeared to emerge multifocally from ancestors that were clonally related but not identified as cancerous. This observation challenges the traditional linear evolutionary model of cancer, which suggests the development of a singular cancer progenitor. Instead, it supports a branching evolutionary pattern where multiple cancer founders may emerge from a non-cancerous population. This pattern appears to be more prevalent during cancer progression than previously recognized [26].

These discoveries underscore the importance of PRE-cancerous states, which formation occurs much earlier than cancers per se.

## 4. Vestiges of Positive Selection in Modern Humans versus Other Species: Mutations in Oncosuppressors and Oncogenes Pertinent to Hereditary Human Breast Cancer

Selection pressures have evidently affected a significant portion of the human BRCA1 protein sequence, particularly the segment encoded in exons 12 to 16. Missense mutations that pose the greatest risk for breast and ovarian cancers are situated in evolutionarily conserved regions, sites of phosphorylation, and within protein-binding domains [27,28,29,30]. Indeed, there are many papers that describe the detection of positive selection signals in BRCA1 and BRCA2 despite the fact that selective pressures on human cancer genes throughout mammalian evolution might not be directly associated with cancer.

Crespi and Summers, in as early as 2006, reviewed hypotheses about positive selection and its effects in BRCA1 and BRCA2 inactivating mutation carriers [30]. During human development, particularly as triggered by puberty, the selective advantages of certain genetic traits are notable. For instance, alleles that promote faster proliferation might be more beneficial to one sex over the other [30]. A broad perspective on the subject has also been presented as a hypothesis: in the course of human evolution, this increase in breast size might have led to the preferential selection of alleles that facilitate rapid breast development. These alleles may also exert antagonistically pleiotropic effects, potentially increasing the risk of BC [30].

Importantly, it was suggested that the region under positive selection (exon 11 and also exons 12–16 in BRCA1) is the RAD51 interacting domain [28].

Among the genes identified as likely undergoing positive selection, there are genes associated with DNA repair, particularly those connected to the Fanconi Anemia (FA)/BRCA pathway, including BRCA2, CHEK2, FANCC, and others. BRCA1 and BRCA2 genes have likely been subject to significant positive evolutionary selection: BRCA2, in the early ancestors of modern humans, and BRCA1, in the evolutionary lineage that included chimpanzees, bonobos, and humans [31,32]. The study on 23 primate species revealed that certain amino acid sites underwent recurrent selection for amino acid replacement throughout primate evolution [32]. Notably, this selection was predominantly observed in humans as well as in our closest extant relatives—chimpanzees (*Pan troglodytes*) and bonobos (*Pan paniscus*). Thus, it became evident that while mutations causing truncated BRCA1 forms are associated with cancer predisposition in humans, there is an inherent selection favoring amino acid-altering substitutions in this gene.

O’Connell noted that approximately one third of the BRCA1 protein is subject to purifying selection and one third is under positive selection. In contrast, he proposed that the majority of BRCA2, about 96%, is experiencing either negative selection or neutral evolution. Only a small portion, around 4%, of the BRCA2 protein is under positive selection. This positively selected segment exhibits a distinct pattern, primarily located at the termini of the protein and between the eight BRCT domains. This pattern may arise from selective pressures favoring enhanced BRCT interactions [33].

Footprinting analysis of the Ka/Ks ratio in the primate BRCA1 gene revealed three distinct regions with ratios exceeding one, suggesting possible positive selection (reported Ka/Ks ratios are summarized in Table 1). Notably, two of these regions are located within the RAD51 binding domain, hinting that changes in the RAD51 interaction might be a key factor in the adaptive evolution of BRCA1 in primates. Furthermore, since the evolutionary divergence between the lineages leading to chimpanzees, bonobos, and humans occurred approximately six million years ago, the human branch has accumulated a notable total of 25 substitutions in the BRCA1 gene. Of these, 22 are nonsynonymous substitutions [34].

BRCA2 exhibits signs of positive selection in other primate species, specifically within the Rad51 interaction domain. However, this trend is not observed in the clade comprising humans, chimpanzees, and bonobos. The domains under selection are known to facilitate interactions with related proteins [32].

Comparison of the unique long exon 11 (long in both BRCA1 and BRCA2, despite a lack of homology between them) in primates versus other mammals revealed that there are certain distinctive features. In Laurasiatheria, only a few characteristics are shared, and there is also one site under positive selection that is shared with Marsupialia. Primates also exhibit a positively selected site at codon 835. Interestingly, within the mammalian group, these regions may be currently experiencing purifying selection in eutherians [35].

In a remarkable contrast, it has been argued that only 35 cancer driver genes associated with 47 cancer types exhibited recent positive selection. This suggests an adaptive evolution of cancer driver genes in both early human evolution and among contemporary human populations. Specifically, for BC, only genes NIN and CUX1 demonstrated signs of positive selection. Yet, when narrowing the focus to positive selection solely in the human lineage, a mere eight cancer driver genes displayed this trait. This discrepancy could be attributed to the fact that many cancer driver genes played adaptive roles in early primate evolution, but lost their significance in the human lineage. Surprisingly, neither BRCA1 nor BRCA2 exhibited signs of positive selection in humans, even though they might have in other primates and in ancestral hominins prior to the gorilla divergence [36].

The most plausible explanation for this descrepancy probably resides in the significant variation in the intensity and direction of selective pressures at various loci of the genes under consideration. Furthermore, the bulk of Alu insertions occurred in ancestral hominoid lineages post the divergence of Hominidae (around 25–14 MYA) and the rhesus macaque. More recent hominoid lineages mainly underwent deletions. With no significant alterations linked to other repetitive sequences, it is inferred that Alu repeats predominantly influenced the evolution of BRCA1 non-coding sequences, marking the BRCA1 gene as a hotspot for both Alu retroposition and recombination [28].

For evolution within the human lineage, interpretations of the available data are sometimes controversial. For example, there is intriguing evidence from Neanderthals. Their BRCA2 gene had three distinct non-synonymous nucleotide substitutions on chromosome 13 [37]. A hypothesis proposes that these changes in Neanderthal BRCA2 might have been favored because of their impact on cognitive abilities, which could potentially account for certain cognitive distinctions between Neanderthals and contemporary humans. However, this raises broader questions regarding the susceptibility of human evolution to variations in cancer-related genes [38].

Mutations in the BRCA1 and BRCA2 genes linked to BC are significantly more prevalent among Ashkenazi Jews than in other populations. Three hypotheses have been proposed to account for the extensive haplotype structures found in Ashkenazi chromosomes with these mutations: endogamy, positive assortative mating, and positive natural selection. However, the theory of positive assortative mating lacks empirical support, failing to correspond with deviations from the Hardy–Weinberg equilibrium. Therefore, this theory can be set aside based on more parsimonious reasoning. Similarly, the hypothesis of positive natural selection is challenged by discrepancies with the broader demographic patterns observed in the population. Consequently, these considerations lead to the conclusion that the high incidence of BRCA1 and BRCA2 mutations in the Ashkenazi Jewish population is more likely attributable to genetic drift than to specific selective pressures. The most plausible explanation appears to be a bottleneck and a founder effect. Indeed, the founder effect aligns with the current knowledge of Ashkenazi demographic history [39].

Yet, it is impossible to completely dismiss the hypothesis that selection played a role because the aforementioned conclusions are based on the reasoning of parsimony. Also, it is important to note that, in humans, sexual selection is not simple assortative mating. Rather, sexual selection is inseparably intertwined with social selection; for example, most attractive women may receive more care, thus securing the best chances for the survival of their offspring. Unfortunately, such hypotheses are difficult to test experimentally.

**Table 1:** Reported K_ns_/K_s_ values for BRCA1, BRCA2, and other genes important for human BC.

Gene, Region	Lineages with Focus on	Other K_ns_/K_s_	K_ns_/K_s_ for Homo	Additional	Ref.
BRCA1, exon 11	Primates	Chimp = 2.6, Gor = 0.4	3.1		[29]
BRCA1, full length	Great apes	Chimp = 0.98, Gor = 0.65	2.54		[28]
BRCA2, full length	Mammals	Mammalian = 0.47		Mean for 430 genes = 0.12	[31]
BRCA1, full length	Mammals		36% have 5.2485		[33]
BRCA2, full length	Mammals		4% have 2.37		[33]
BRCA1, full length	Primates		Two sites > 2.5 in RAD51 domain	K_ns_/K_s_ footprinting	[34]
BRCA1, full length	Primates	Chimp = 2.66	2.79		[32]
BRCA2, exon 11	Primates		0.24		[32]
BRCA1, exon 11	Mammals, 132 species				[35]

Chimp—*Pan paniscus*, Gor—*Gorilla*. Note that mutation models use different assumptions about the importance of sequence features; therefore, K_a_ and K_s_ estimations may be slightly different between studies.

A leading hypothesis attributes this rapid BRCA1 gene evolution to viral infections. Many viruses interact with DNA repair proteins to stealthily infect their hosts. Viral DNA can activate the DNA damage response and successful infections necessitate viral suppression of this reaction. Some viruses also harness DNA repair proteins for replication. It is plausible that such viruses engage with BRCA1 or BRCA2, and that natural selection could drive gene alterations that reduce susceptibility to these viruses [40]. However, it is doubtful that this logic may be applied to the genesis of inactivating mutations in BRCA1 or BRCA2. For example, in the case of bacterial infections, as exemplified by *H. pylori*, such mutations confer increased cancer risks without any observable benefits [41].

## 5. The Enigma of Ubiquitous Expression of Genes Associated with Breast Cancer Risk like BRCA1 and BRCA2

Obviously, yet intriguingly, tissue-specific genes tend to be more suppressed in cancer compared to their normal counterparts, while non-tissue specific genes are typically activated. This pattern is consistent across 21 analyzed cancer types [42]. The mammary gland is a highly specialized tissue; therefore, BC cells may lack many features specific for normal breast epithelial cells.

**Figure 1:**
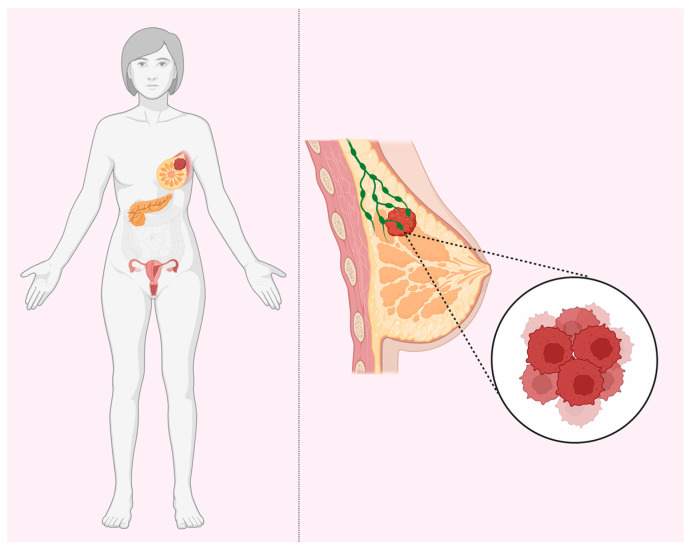
BRCA tissue-specificity paradox. BRCA1 and BRCA2 haploinsufficiency increases the risks for breast, ovarian, and pancreatic cancers despite ubiquitous expression in all tissues.

Several prominent authors published a number of reviews on the hypotheses that may be suitable for the explanation of the remarkable tissue specificity of early cancers (HBOC) in BRCA1 and BRCA2 heterozygous carriers (Figure 1). The question is why these mutations (in BRCA1 and BRCA2, etc.) yield increased risks of cancer in a tissue-specific manner (breast, ovary, and to a much lesser extent, pancreas, prostate, etc.), whereas the genes themselves, both BRCA1 and BRCA2, are expressed ubiquitously, with any predilection for certain tissues. Schaefer, Serrano, and others [43,44,45,46,47,48] made a synthesis of different ideas and classified various mechanistic explanations into major potential types, most of which are, philosophically, speculations on the presence or absence of unknown factor(s) in the breast and ovary tissues.

(1) Variations in DNA repair mechanisms, as well as the rate of loss of heterozygosity (LOH), may exist across different tissues. It is possible that some tissues have proteins that can compensate for the lack of BRCA1 function, particularly in terms of DNA repair. If this is the case, breast and ovarian cells might uniquely lack this compensatory mechanism, making the absence of BRCA1 more impactful in these tissues compared to others that have such support systems. Nevertheless, the occurrence of significant embryonic defects in BRCA1 knockout mice suggests that this idea of redundancy might not be entirely accurate.

(2) Due to high proliferative activity inherent to breast and ovarian tissues, absent BRCA1 in such environments could boost mutation rates, subsequently increasing the chances of cancer mutations. While plausible, this hypothesis becomes questionable when considering other tissues, like the colon, which exhibit high proliferation but not the same heightened tumor formation.

(3) The full absence of BRCA1 typically results in cell death or markedly reduced proliferation in most tissues. But in BC and OC, cells devoid of BRCA1 can survive—for some reason much longer—accumulating secondary mutations that foster cell growth. The underlying reason is not necessarily DNA repair redundancy across tissues. Instead, only breast and ovary cell cancers might have the tenacity to survive without BRCA1 for extended durations. Such endurance could stem from unique genetic factors, including additional DNA repair capabilities or the expression of BRCA1-interacting proteins that counterbalance certain lost functions.

(4) Another possibility is the higher expression levels of anti-apoptotic genes, which deter DNA damage-induced cell death. Furthermore, the physiological environment of these tissues might also play a role. However, the fact that a majority of BRCA1 tumors do not express these receptors complicates this theory.

(5) Breast and ovarian cells might have an apoptosis delay compared to other rapidly dividing cells. Consequently, these tissues might tolerate the loss of the wild-type (WT) BRCA1 allele for a more extended period. Yet, no empirical studies corroborate this notion, leaving the actual delay in apoptosis in these cells ambiguous.

(6) Estrogen exposure amplifies DNA damage and genetic instability, heightening the risk of BRCA1-associated BC and OC (HBOC). BRCA1 and 2 seemingly safeguard breast epithelial cells from oxidative DNA damage, which results from elevated reactive oxygen species, a consequence of hormone-induced growth and its cellular metabolic impact. If DNA lesions from this oxidative damage proceed into the S-phase, it leads to double strand breaks, necessitating BRCA1/2-mediated homologous recombination (HR) repair. This theory is appealing, yet it is puzzling that other hormone-sensitive tissues (like the uterus, cervix, bone marrow, and brain) do not manifest increased cancer rates in BRCA1 mutation carriers. This poses the question: why would estrogen metabolism differ solely in breast and ovarian tissues?

(7) The breast and ovary respond to estrogen, perhaps simply more sensitively than most aforementioned tissues. Some estrogen byproducts can bind to DNA, potentially acting as tissue-specific carcinogens. This phenomenon, termed “remote carcinogenesis”, could be intensified by a BRCA mutation, especially if the associated DNA repair pathways are compromised [49]. Therefore, considering non-cell-autonomous mechanisms, it is important to note that intercellular interactions significantly impact both monogenic and polygenic conditions. A key factor influencing tissue-specific functions is the differential responsiveness of various tissues to certain signals. This is often mediated by tissue-specific proteins, which frequently function as receptors. A pertinent example of this phenomenon is observed in the case of BC and OC that arise due to familial mutations in the BRCA1 gene. Both the breast and ovarian tissues prominently express ER, making them responsive to estrogen. Estrogen signaling can provoke DNA double-strand breaks in BC cells, which in turn leads to the formation of pathological topoisomerase II–DNA complexes. In a normal physiological context, where BRCA1 is functional, these complexes are efficiently resolved. However, in cases where BRCA1 functionality is impaired, such as in certain familial cancers, these complexes accumulate [50].

(8) BRCA gene haploinsufficiency might elevate the risk of accruing additional cancer-inducing mutations during breast development, even potentially mutating the BRCA gene itself. If puberty presents a unique vulnerability period for BRCA mutation carriers, this might explain the relative scarcity of BRCA gene inactivation in sporadic breast or ovarian cancers.

(9) The role of the microenvironment cannot be overestimated. Indeed, epithelial stem cells already received signals from the microenvironment during tissue (breast) development. Conditional BRCA1 knockout (KO) experiments in mouse models underscore the significance of BRCA1 in mammary gland development. Also, they may continue to receive signals like ROS, eicosanoids, etc., throughout the lifespan.

(10) The susceptibility of certain tissues to disease could potentially be linked to the reduced expression of the relevant gene. In the case of human mammary epithelial cells (HMECs) with a heterozygous mutation in the BRCA1 gene (BRCA1mut/+), a unique pattern emerges compared to other cell types. These mutations lead to a notable decrease in the expression of SIRT1, a protein associated with cellular longevity and genomic stability. This reduction in SIRT1 expression results in accelerated telomere shortening and increased genomic instability [51].

(11) Some theoretical models propose that primarily mammary stem cells have the longevity necessary to accumulate the multiple genetic alterations required for the initiation of breast tumorigenesis. It is estimated that the population at risk in each cellular niche consists of about 10 stem cells. This number corresponds to the stem cells located in small ducts near the terminal lobules of the breast, amounting to a total of approximately 100,000 cells across the organ. When considering the role of mammary progenitor cells in addition to stem cells, these cells are thought to divide approximately once per day. The hemizygous loss of one BRCA1 allele might initially seem to reduce the cells fitness compared to the homozygous loss of another tumor suppressor gene (TSG). This disadvantage might be mitigated if a second mutation in BRCA1 occurs before the inactivation of the first allele of a TSG. This is supported by the findings showing that cells with both mutated BRCA1 alleles and a hemizygous loss of TSG alleles demonstrate greater fitness than cells with both mutated BRCA1 alleles and intact TSG alleles [52].

(12) Mechanistically, the RANK-RANKL pathway, pivotal for mediating paracrine actions in luminal homeostasis, shows atypical activation in BRCA1 mutation carriers even before evident clinical manifestations of BC. This begs the question of why such an expression is limited to specific tissues. If BRCA1 is abnormally expressed in T cells, it could lead to irregular transcription tied to anti-tumor immunity. Such a scenario could allow for breast tumor cells with BRCA1 mutations to evade the immune system, elevating the BC risk in women with BRCA1 mutations [53].

It should be noted here that a recent study reported a significant increase in gastric cancer incidence in both BRCA1 and BRCA2 mutation carriers in the presence of *Helicobacter pylori* [41]. This finding illustrates that infections can greatly affect tissue-specific patterns of carcinogenesis.

## 6. Peto-like Paradox, Association between Breast Size and Breast Cancer Risk, and Other Factors

Larger animals do not succumb to cancer at higher rates than their smaller counterparts, a phenomenon referred to as the Peto paradox. This has led to two key predictions rooted in the idea of natural selection shaping cancer-related genes.

**Figure 2:**
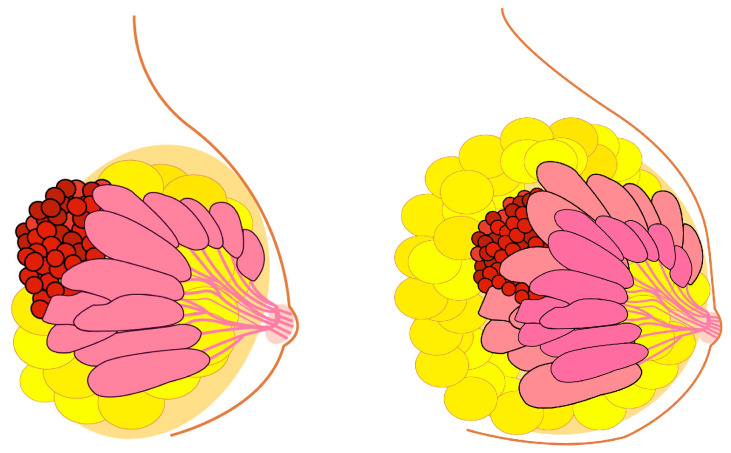
**Breast cancer risk and the enigma of breast size versus breast cancer incidence similar to Peto paradox.** Theoretically, larger breast size should indicate an increased breast cancer risk. This is true, but in a surprisingly small degree.

In line with the reasoning established in the initial definition of the Peto paradox for whole organisms, the relationship between breast size and BC risk presents an extremely interesting case for advancing our understanding of tissue-specific carcinogenesis. Following this Peto-like logic, it would stand to reason that larger breasts could be associated with a higher risk of BC (Figure 2). It can be established that BC incidence is higher in *Homo sapiens* than in *Pan* (chimpanzees and bonobos), aligning with the initial logic of the Peto paradox.

Early studies examining the correlation between breast size and BC risk yielded inconclusive results. An initial review of ten studies generally found no associated risk [54]. In general, the literature on the direct role of breast size in relation to BC has been varied in conclusions. Among the 20 reviewed papers, the findings were inconsistent and sometimes contradictory [55]. When specifically considering brassiere size, only a minor positive correlation was observed [56]. A study involving a large number of Caucasian women observed a slight inverse relationship between breast size and BC risk. The odds ratio (OR) was 1.37 for the smallest brassiere size compared to the largest. However, after adjusting for the main recognized BC risk factors, this increased risk disappeared, resulting in an OR of 1.16 for brassiere sizes ≤1 compared to ≥5. Furthermore, no significant variations in BC risk based on breast size were observed across various factors such as age at diagnosis, parity, and family history of BC, among others. Hence, this comprehensive study affirmed that there was no significant link between breast size and BC risk in this particular Italian population [57]. It is essential to interpret these results with caution, since bra cup size is not a reliable indicator of glandular mass since adipose tissue can constitute up to 80% of a woman’s breast. Moreover, plastic surgeons are aware that many women incorrectly select their bra size [58].

Subsequent research indicated a slight yet clearly positive relationship, where the baseline bra cup size emerged as the strongest predictor of BC mortality. The association between bra cup size during youth and the likelihood of developing premenopausal BC has been found, especially in leaner women [59].

Thus, when considering breast size before pregnancy, it emerges as a positive predictor for postmenopausal BC. However, this link seems specific to women who were notably lean during their younger years. From the data collected, it appears that young women who had larger breasts faced a heightened risk of BC. However, this observation might be muddled by various factors. Smaller breast sizes might correspond to denser mammographic patterns, which are deemed high risk. Variability stemming from inaccurate recollections or the absence of standardized bra size measurements could further weaken any positive associations [60].

In the whole organism, the within-species relationship between size and cancer risk is clearer. For instance, taller individuals have a higher risk of developing melanoma. However, this correlation becomes less obvious when examining three-dimensional organ structures. It becomes apparent only after substantial methodological improvements, including adjustments for factors like adipose tissue. This slow change in the understanding marks a shift from initially denying any correlation to recognizing and affirming its existence. This phenomenon where increased cancer risk correlates with larger organ size is evident in lean females, in other words, the Peto-like paradox appears to be absent in lean females. The adipose tissue conceals this relationship.

Breast size and BC share some genetic underpinnings, though the relationship is quite subtle. A Genome-Wide Association Study (GWAS) revealed that certain genetic variations in shared regions are associated with both breast size and BC. Specifically, two single nucleotide polymorphisms (SNPs) located near ESR1 and PTHLH have been linked to breast size and have also been previously tied to BC risk [61].

Risk-reducing mastectomy decreases the risk of BC development, and risk-reducing salpingo-oophorectomy decreases OC-specific as well as overall mortality [62]. More specifically, bilateral prophylactic mastectomy, often termed bilateral risk-reducing mastectomy, is the predominant risk-reducing surgery. For women over 40, breast reduction surgery correlates with a subsequent decreased BC risk. Especially in the context of familial cancer, the protective benefits of the surgery are substantial. Prophylactic mastectomy leads to at least a 90% decrease in BC incidence for those with a family history of the disease [63,64].

## 7. The Case of Male Breast Cancer

Men represent about 1% of all BC diagnoses in the USA. Of these, 97% of the tumors were ER-positive, 92% were PR-positive, and 16% were HER2-positive. Typically, men diagnosed with BC in the USA are 5–10 years older than women. Potential risk factors deserving further investigation include mutations in PTEN, CYP17, and CHEK2, as well as environmental exposures. Approximately 3 to 14% of male BC cases, especially those with a family history, can be attributed to BRCA2 mutations [65,66,67].

## 8. Fertility and Breast Cancer Predisposition

The fertility data concerning female BRCA carriers present mixed findings. Some studies suggest there is no decline in fertility among female BRCA carriers, or there is even a slight fertility uptick among BRCA1/2 mutation carriers living in natural fertility settings, despite increased post-reproductive mortality compared to non-carriers [68]. Interestingly, despite these findings, BRCA1 mutation carriers exhibit a diminished ovarian response to stimulation, resulting in fewer mature oocytes, a trend not seen in BRCA2 mutation carriers [69]. Rather interestingly, there is evidence that both female and male BRCA mutation carriers have higher fertility and overall fitness in comparison to non-carriers from the same families [70].

When considering the effects of pregnancy, BRCA1 mutation carriers do not exhibit a significant link between BC risk with parity relative to nulliparity. Conversely, for BRCA2 mutation carriers, being parous corresponds with a 30% increase in BC risk, with no discernible risk reduction observed for multiparous women [71]. Notably, breastfeeding for at least a year appears to diminish BC risk for BRCA1 carriers, while no such effect has been identified for BRCA2 carriers [72,73].

## 9. Hormonal Status and Breast Cancer—Is There a Link with Putative Sexual Selection?

Throughout the lifespan, mammary epithelium experiences dynamic changes in sync with menstrual cycles, pregnancy, childbirth, and subsequent breastfeeding. These changes contribute to its unique mutation profile. Notably, there is a marked reduction in mutation rates after menopause, potentially due to diminished cell turnover from the cessation of menstrual cycles and decreased estrogen levels. Moreover, pregnancy and childbirth, which pause menstrual cycles, may counteract mutation accumulation. This aligns with epidemiological findings that suggest a connection between late menopause and low parity with an increased risk of BC. A reduction in BC risk was observed in association with ovarian ablation for ovarian cysts among those with BRCA1 and BRCA2 mutations [74]. The protective effect against BC observed in BRCA1 carriers after ovariectomy may stem from the removal of paracrine signals from differentiated ER/PR-positive luminal cells directed towards primitive stem/progenitor cells [21].

There is a consensus suggesting that hormonal cycles play a role in BC. Notably, in situations with genetic or transgender individuals who have typical female breasts and estrogens but lack progesterone, the BC incidence is notably low [75]. This underscores the pivotal role of progesterone in the disease’s etiology.

A study conducted by Goldberg and colleagues in 2017 reported higher androgen levels in BRCA1/2 pathogenic variant carriers compared to non-carriers [76]. This led to the famous speculation that BRCA mutations might represent a case of antagonistic pleiotropy, where mutations confer benefits during the reproductive bloom at the cost of detriments in old age. This could manifest as a reduced female lifespan versus enhanced male fitness, perhaps together with a sexual conflict, exemplified here with increased male fitness and increased BC incidence in females. Unfortunately, this intriguing hypothesis lacks broader support, as more recent studies on larger cohorts indicate that male hormone levels remain relatively consistent [77]. Nonetheless, this hypothesis might still hold merit when specifically examining the Jewish population, despite the ready explanation that BRCA1 and BRCA2 frequencies in Ashkenazi Jews can be adequately explained via a historical bottleneck event, rendering it unnecessary to invoke positive selection [39].

Another captivating hypothesis suggests that sexual selection drove significant changes in breast size and shape in humans after diverging from the *Pan* genus (chimpanzees and bonobos). These morphological changes may have been connected to evolutionary trade-offs, including modifications to BRCA1 and BRCA2 genes. These changes could have rendered the respective proteins more susceptible to mutations. However, these theories might only be thoroughly validated with genetic experimentation in chimpanzees. Moreover, a substantial body of recent evidence suggests that pathogenic mutations in the BRCA genes have emerged relatively recently in human evolutionary history [78]. We should reiterate here that the most likely proposition for this rapid evolution is that it is an offshoot of an evolutionary arms race against viral infections. Hence, antiviral immunity could be tied to the observed positive selection. Supporting this idea is the observation that HIV specifically targets BRCA1 in nuclear loci, co-opting DNA repair mechanisms for its replication [79].

The role of sexual selection in understanding cancer biology has long been proposed. However, the idea previously lacked specific details and saw limited progression [80]. Again, it is important to note that male carriers of BRCA1/2 pathogenic variants (PVs) displayed an androgen profile similar to that of non-carriers. Furthermore, hormonal levels did not show a significant correlation with the incidence of prostate cancer (PCa) in men, regardless of their BRCA1/2 PV status. This suggests that the aggressive phenotype of PCa observed in BRCA2 PV carriers may not be linked to variations in circulating hormone levels. It is also noteworthy that the average hormone levels for both carrier and non-carrier groups fell within the normal physiological range [77].

## 10. The Importance of Understanding Evolutionary Aspects of Breast Cancer Genesis for Breast Cancer Prevention and Treatment

In a mouse model, early signs of genome instability were evident in BRCA1 heterozygotic mutants [81] as early as the 10.5-day embryo stage, characterized by structural variations, indels, and copy number variations. Intriguingly, this instability affected numerous oncogenic genes and pathways, spanning DNA damage repair, estrogen signaling, and oncogenesis. Furthermore, a mutation in p53 was not a prerequisite for genome instability spurred by a BRCA1 mutation in non-cancerous cells. A heterozygous BRCA1 mutation was sufficient to induce genome instability during embryogenesis [81]. This finding indicates that humans, in comparison to mice, seem more resistant to BRCA1 haploinsufficiency, underscoring the presence of some specific anti-cancer mechanisms in humans that are absent in mice. On the other hand, the potential necessity of prevention timelines for BRCA1 mutation-associated cancers in humans should be reconsidered, suggesting earlier interventions than currently practiced.

### Molecular Mechanisms behind Preventive Strategies for BC

Although BRCA haploinsufficiency inherently increases the risk of “geroncogenic” events, it also makes patients more susceptible to both preventive and therapeutic strategies, which utilize novel drugs or approaches designed to counteract the aberrant metabolic reprogramming observed in mammary/ovarian epithelial cells. Apart from well-known drugs targeting PARP, there are other targets, such as MEPCE, which show promise in the context of BRCA1 deficiency. MEPCE functions to stabilize the 7SK snRNP, which in turn sequesters P-TEFb to modulate the transcription elongation ref. When MEPCE levels are diminished, any transcriptional stress and subsequent DNA damage occurring at transcriptionally active chromatin regions are repaired via homologous recombination (HR). In the absence of BRCA1, a decrease in MEPCE leads to the accumulation of R-loops, transcriptional stress, and conflicts between transcription and replication processes. These disturbances result in DNA damage, creating a condition of synthetic lethality in BRCA1-deficient cells [82].

A distinct behavior between normal cells and those undergoing specific disturbances, such as depletion of SETX/BRCA1 or other pathological conditions impacting R-loop regulation, have been observed. For example, in normal wild-type cells, nuclear R-loops are efficiently resolved by RNase H or RNA-DNA helicases like SETX. A small proportion of R-loops are processed via XPG, leading to the formation of cytoplasmic RNA-DNA hybrids. However, the concentration of these hybrids remains below the level required to initiate IRF3 signaling activation. In perturbed or malignant cells, some nuclear R-loops may not be resolved effectively. These unresolved R-loops are processed via XPG, resulting in an increased accumulation of RNA-DNA hybrids in the cytoplasm. These hybrids are detected via cGAS and TLR3 in the cytosol and endolysosomes, respectively, triggering the activation of IRF3-mediated immune signaling and apoptosis [83].

The current list of possible chemopreventive interventions includes estrogen-receptor modulator (SERM) tamoxifen, aromatase inhibitor exemestane, poly (ADP-ribose) polymerase inhibitors veliparib and Olaparib, and perhaps also the RANK ligand inhibitor denosumab. Recent discoveries open the way to rethinking the strategy of prevention and treatment of BC. Indeed, since BRCA-related cancers preferentially affect estrogen target organs, it is conceivable that hormones acting as survival factors may protect BRCA-deficient cells. Mouse models of BRCA1 and BRCA2 provide an opportunity to further explore these hypotheses [11,43].

The menstrual cycle in humans, similar to the estrous cycle in mice, is a significant risk factor for the development of both mammary and serous ectopic Müllerian carcinomas. Disruptions of this cycle have shown a protective effect against these cancers even in individuals with a genetic predisposition, including BRCA1 mutation carrier. Hypothetically, the menstrual cycle in BRCA1 mutation carriers may be particularly sensitive to olfactory ligands. Hence, it is worth investigating whether targeting specific olfactory agents can effectively reduce cancer risk in young BRCA1 mutation carriers who wish to postpone risk-reducing surgery to preserve fertility [84].

## 11. Conclusions

A plethora of hypotheses aimed at both the prevention and treatment of BC can be conceived, extending beyond the option of mastectomy. Yet, the empirical validation of these hypotheses poses a formidable obstacle. The exigency for life-long studies to ascertain accuracy further amplifies the complexity of this challenge. However, incorporating an understanding of evolutionary factors, especially those pertaining to natural and sexual selection, proves instrumental in the judicious selection of research hypotheses. This nuanced perspective serves a dual purpose: it not only facilitates cost-efficiency but also accelerates the trajectory towards transformative breakthroughs for BC prevention and treatment.

On an even broader evolutionary scale, it appears that, in contrast to other great apes, certain cancers are uniquely prevalent in humans, including lung, prostate, and testicular cancer [85]. This observation of distinct cancer patterns across different species emphasizes the importance of cross-species comparative studies in understanding cancer biology. Moreover, this observation reinforces the need for a comprehensive comparative study of breast tissue in chimpanzees and bonobos versus humans. Since many standard laboratory experiments typically conducted on animals like mice are impossible with great apes, alternative approaches such as transcriptomic and epigenetic studies, particularly in vitro, are warranted.

From a mechanistic perspective, the human KIT protein kinase likely plays a pivotal, yet concealed, role. However, when addressing familial BC in carriers of BRCA1 and BRCA2 mutations, a distinct approach is required, emphasizing developmental influences persisting over the individual’s lifespan and continuously impacting the affected cells.

Additionally, the mechanisms in familial BC might involve proteins interacting with BRCA1 and BRCA2. Another hypothetical mechanism could involve unresolved R-loops in mutation carriers, leading to chronic activation of the cGAS-STING nucleic acid sensing pathway (reviewed, for example, in [86]). Normally, this pathway is a potent oncosuppressor when it is excited in an acute mode, while its chronic activity can cause overstimulation of surrounding tissues and immune cells by interferons, leading to tachyphylaxis and continuous non-canonical NF-kB pathway activation in malignant cells. Thus, impaired R-loop resolution may be linked to immune suppression and an increased dissemination of early tumors.

These insights suggest the existence of significant oncosuppressive adaptations in human breast tissue, laying a foundation for future advancements in this field. Analogous to the multiple copies of the p53 gene in elephants [87,88], which confer enhanced cancer resistance, similar mechanisms might be present in humans.

If the human mammary gland indeed has some human-unique and tissue-specific anti-carcinogenesis mechanisms, then it is logical to propose that these may be particularly vulnerable in the context of whole-organism anomalies like haploinsufficiency of BRCA1 and BRCA2. This vulnerability arises because the tissue depends on recent, but relatively fragile, evolutionary developments that, while beneficial, are not sufficiently robust as they do not extend to the organism as a whole—in contrast with the global influence of BRCA1 or BRCA2 haploinsufficiency.

The significance of non-cell-autonomous processes cannot be overlooked. These processes might operate at a systemic level, as observed in hormone-producing tissues like the ovaries, or in adjacent tissues such as adipocytes or fibroblasts. The microenvironment also influences cells with BRCA1 and BRCA2 haploinsufficiency.

However, it is crucial to recognize that sporadic and familial BC likely follow different mechanisms and evolutionary trajectories and thus should be studied separately to understand their unique dynamics and potential treatments.

## Data Availability

Not applicable.
